# Supplementary material for: The 22q11.2 region regulates presynaptic gene-products linked to schizophrenia
Source: Nat Commun. 2022 Jun 27;13:3690. doi: 10.1038/s41467-022-31436-8 (PMC9237031; doi:10.1038/s41467-022-31436-8)
Supplement: Supplementary file 1 — Supplementary Information [file 41467_2022_31436_MOESM1_ESM.pdf]

Supplementary Information

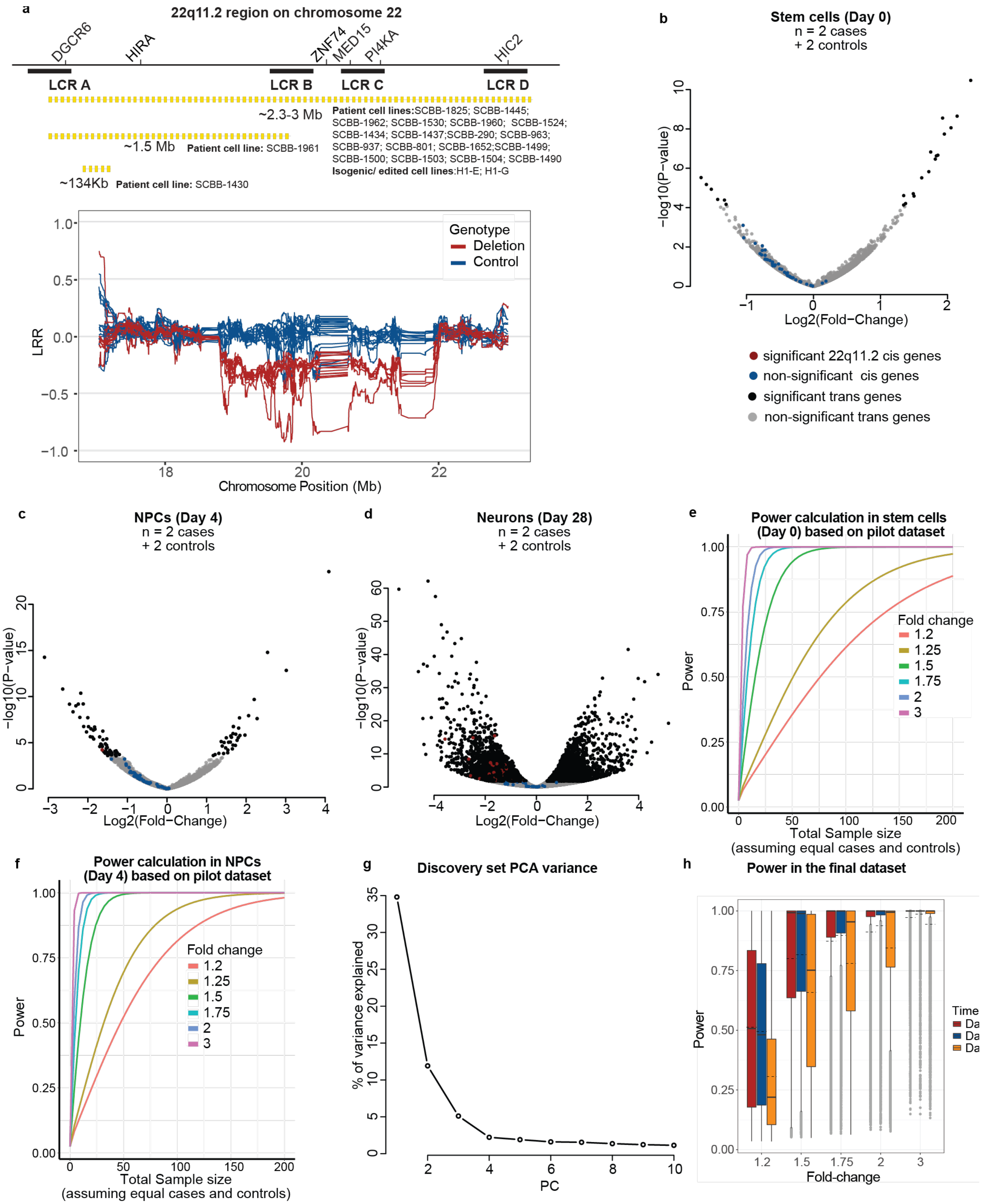

Supplementary Fig. 1. Discovery and pilot datasets. a, Top: Schematic of the 22q11.2 region, including the low copy repeats (LCRs). The breakpoints identified in each of the cell lines carrying a deletion in 22q11.2 (listed in Table 1) are shown. Bottom: Validation of the full-size deletion in 22q11.2 lines used in the current study by sliding-window average of SNP marker intensity (LRR) in the deletion locus. **b-d**, Volcano plots showing differentially expressed genes in the pilot dataset (Wald-test from DEseq2, N (cases) = 2, N(controls)=

2). **e**, Power estimation in the pilot dataset for median expressed genes for different fold-changes and sample sizes in stem cells. **f**, Power estimation in the pilot data set for median expressed genes for different fold-changes and sample sizes in neuronal progenitor cells (NPCs). **g**, Variance explained by the first 10 principal components in the discovery sample. **h**, Estimated power in the final (discovery) dataset, at each time point (N(patients) = 20 , N(controls) = 29). The power estimates are presented in a Tukey style boxplot with the median (Q2) and the first and the second quartiles (Q2, Q3) and error bars defined by the last data point within +/- 1.5-times the interquartile range.

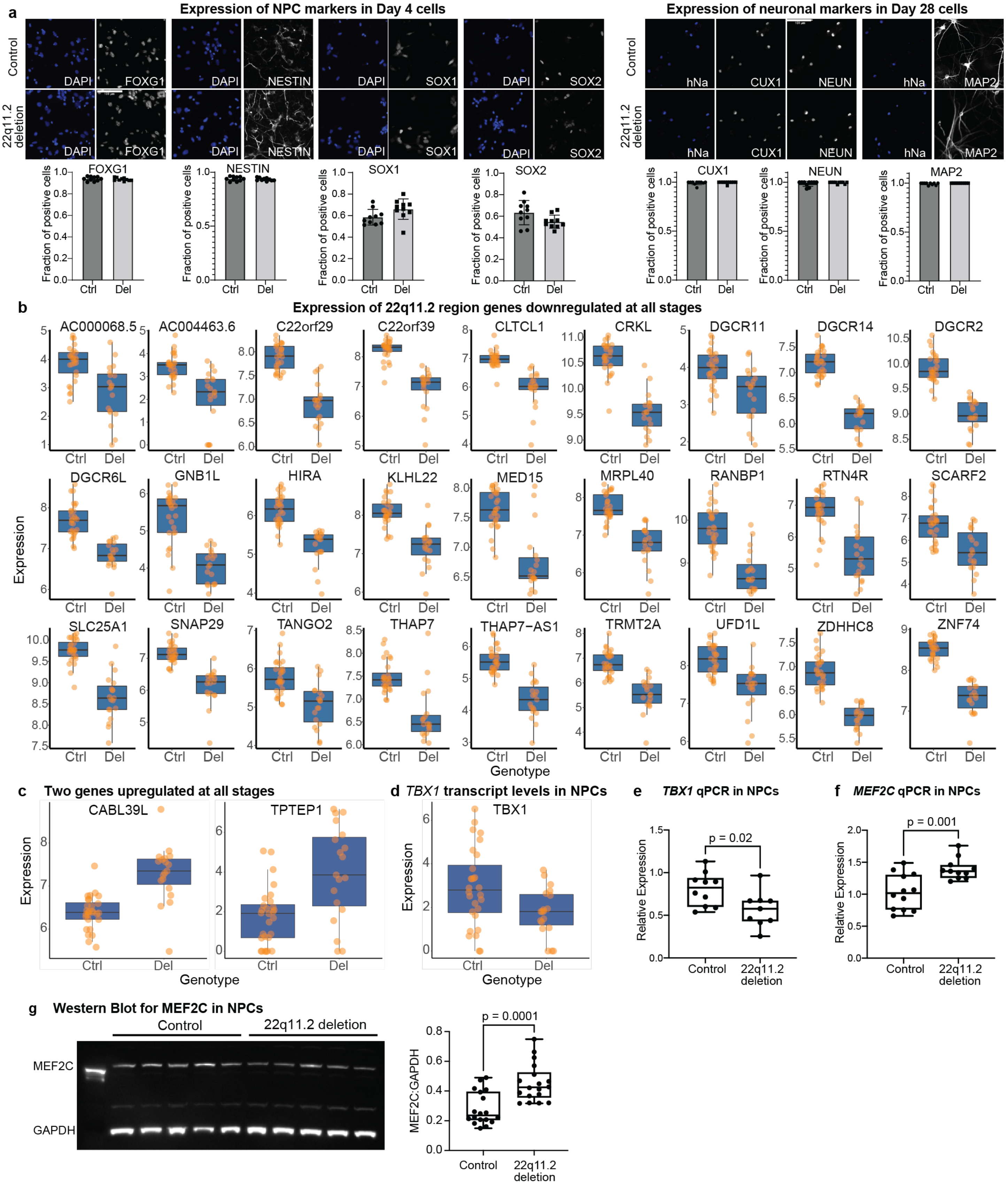

Supplementary Fig. 2. Expression of differentially regulated genes. a, Left, Expression of NPC markers. Representative images of cells labeled with DAPI (blue) and SOX1, SOX2, FOXG1 and NESTIN (white). Quantification of the proportion of cells labeled with each marker to the DAPI+ nuclei is shown. Scale bar is 100 µm. N=10 independent cell lines/condition. FOXG1: Control 0.94, Case, 0.93, p=0.57. NESTIN: Control 0.85, Case 0.87, p = 0.30. SOX1: Control 0.59, Case 0.66, p=0.062. SOX2: Control 063, Case 0.55, p = 0.052,

Student's t test. Right, Expression of mature neuronal markers. Representative images of D28 neurons plated on glia labeled with human nuclear antigen (hNA) to differentiate neurons from mouse glial cells. Scale bar is 100 µm. Quantification of the proportion of cells labeled with each marker to the hNA+ nuclei. CUX1: Control 0.99, Case 1, p=0.49. NEUN: Control 0.99, Case 1, p=0.26. MAP2: Control 0.99, Case 1, p=0.08, Student's t test. Data are presented as mean values +/- SEM. **b**, Expression in D28 neurons of significant cis genes shared across all three developmental stages. **c**, *CAB39L* and *TPTEP1* are the only trans genes upregulated in all stages (N (patients) = 19, N (controls) = 29). **d**, *TBX1* is downregulated in NPCs of 22q11.2 deletion carriers (N (patients) = 19, N (controls) = 29). **e,** Relative expression of *TBX1* via qPCR in Day 4 NPCs from control and 22q11.2del patients (N=6, p=0.02). **f**, Relative expression of *MEF2C* via qPCR in NPCs from control and patients (N=6, p=0.001). **g**, Expression of MEF2C in total protein lysates from control and 22q11.2del NPCs. (Left) Total protein lysates from control (left five lanes) and deletion lines (right five lanes) probed for MEF2C (top) and GAPDH (bottom). (Right) Statistical analysis by two-sided Student's t test reveals statistically significant increase in MEF2C expression in the deletion lines. (N=10, p=0.0001). For each boxplot in b-g the data is presented in a Tukey style boxplot with the median (Q2) and the first and the second quartiles (Q2, Q3) and error bars defined by the last data point within +/- 1.5-times the interquartile range.

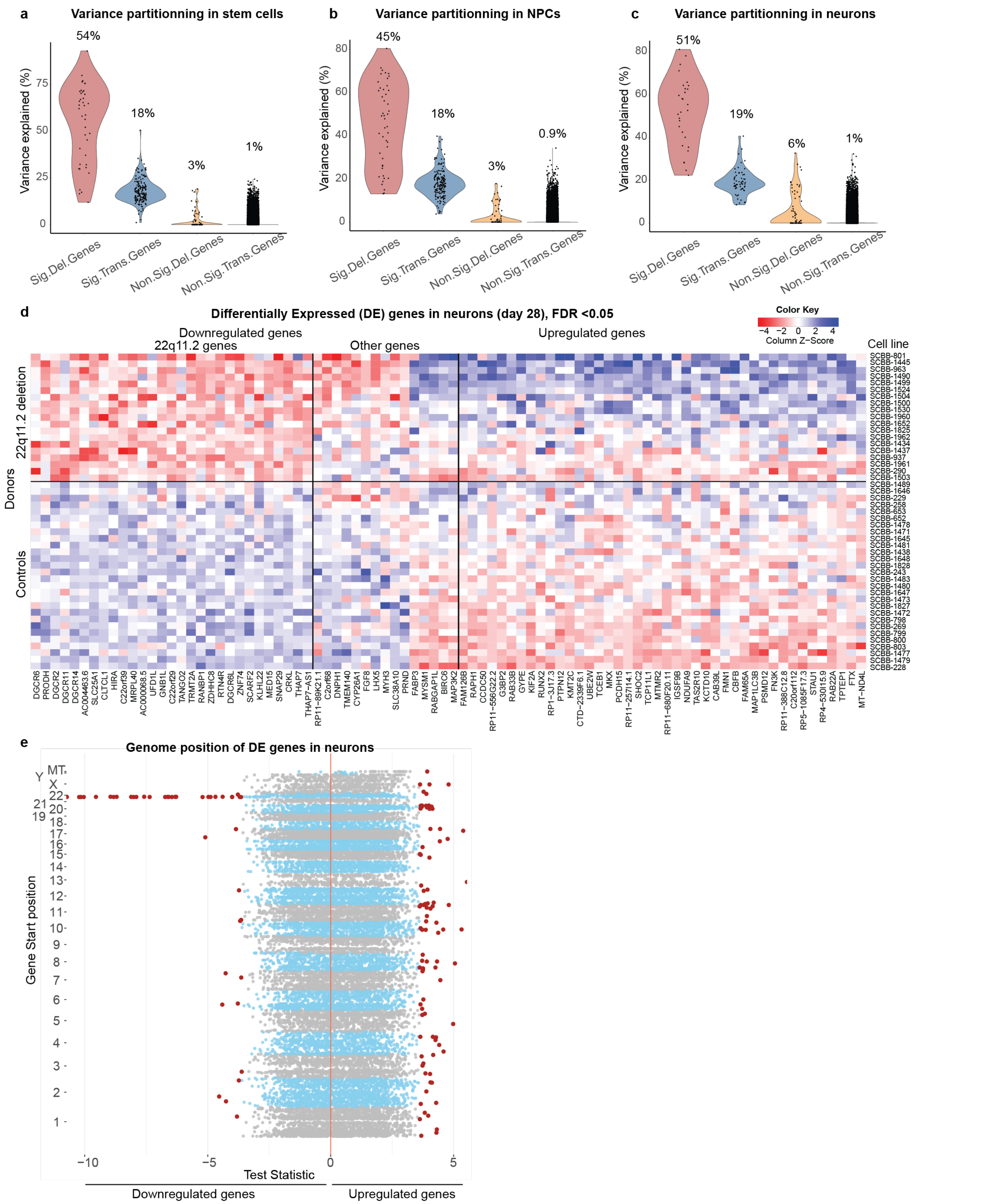

Supplementary Fig. 3. Variance partitioning and expression of differentially regulated genes a-c, Variance in gene expression explained by the deletion genotype in different gene categories in the final dataset in **a**, Stem cells, **b**, Neuronal progenitor cell-like and **c**, Neurons. **d**, Heatmap of 133 genes differentially expressed in neurons showing the range of expression, in all donor lines, of genes down or upregulated. **e**, Test statistic for differential expression plotted by chromosomal position of differentially expressed genes in cells with 22q11.2 deletion. Differentially expressed genes (FDR<5%) are colored in red.

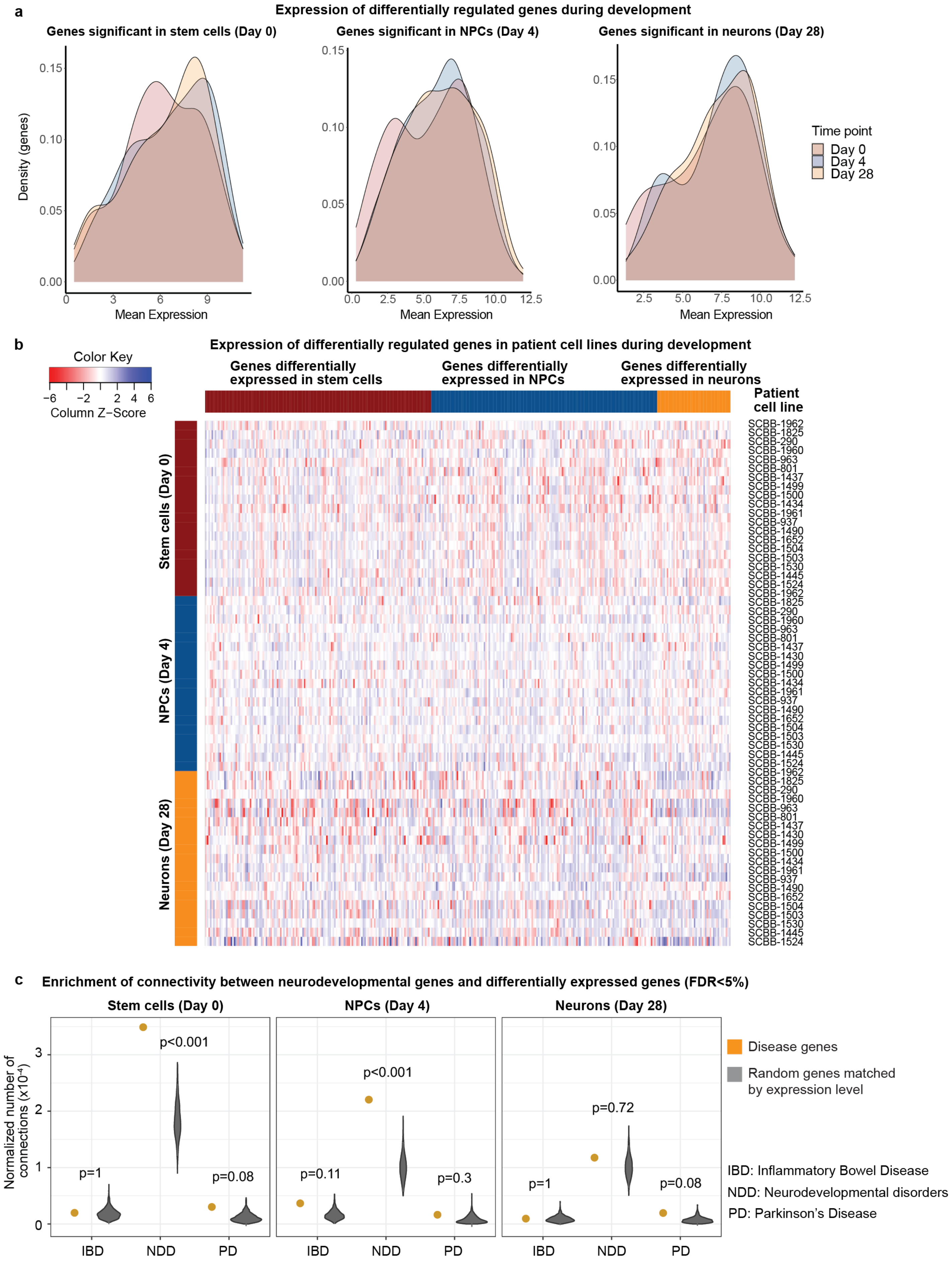
a
Expression of differentially regulated genes during development
Genes significant in stem cells (Day 0)
Genes significant in NPCs (Day 4)
Genes significant in neurons (Day 28)
Density (genes)
0.15
0.10
0.05
0.00
0
3
6
9
0.0
2.5
5.0
7.5
10.0
12.5
Mean Expression
Time point
Day 0
Day 4
Day 28
b
Expression of differentially regulated genes in patient cell lines during development
Color Key
−6 −2 2 4 6
Column Z−Score
Genes differentially expressed in stem cells
Genes differentially expressed in NPCs
Genes differentially expressed in neurons
Patient cell line
Stem cells (Day 0)
NPCs (Day 4)
Neurons (Day 28)
SCBB-1962
SCBB-1825
SCBB-290
SCBB-1960
SCBB-963
SCBB-801
SCBB-1437
SCBB-1499
SCBB-1500
SCBB-1434
SCBB-1961
SCBB-937
SCBB-1490
SCBB-1652
SCBB-1504
SCBB-1503
SCBB-1530
SCBB-1445
SCBB-1524
SCBB-1962
SCBB-1825
SCBB-290
SCBB-1960
SCBB-963
SCBB-801
SCBB-1437
SCBB-1430
SCBB-1499
SCBB-1500
SCBB-1434
SCBB-1961
SCBB-937
SCBB-1490
SCBB-1652
SCBB-1504
SCBB-1503
SCBB-1530
SCBB-1445
SCBB-1524
SCBB-1962
SCBB-1825
SCBB-290
SCBB-1960
SCBB-963
SCBB-801
SCBB-1437
SCBB-1430
SCBB-1499
SCBB-1500
SCBB-1434
SCBB-1961
SCBB-937
SCBB-1490
SCBB-1652
SCBB-1504
SCBB-1503
SCBB-1530
SCBB-1445
SCBB-1524
c
Enrichment of connectivity between neurodevelopmental genes and differentially expressed genes (FDR<5%)
Stem cells (Day 0)
NPCs (Day 4)
Neurons (Day 28)
Normalized number of connections (x10⁻⁴)
0
1
2
3
p=1
p<0.001
p=0.08
p=0.11
p<0.001
p=0.3
p=1
p=0.72
p=0.08
IBD
NDD
PD
Disease genes
Random genes matched by expression level
IBD: Inflammatory Bowel Disease
NDD: Neurodevelopmental disorders
PD: Parkinson's Disease

Supplementary Fig. 4. Expression of genes differentially regulated at specific cell stages in 22q11.2 deletion cells across developmental stages. a, density plots and **b**, heatmap showing that the differentially regulated genes are expressed at similar levels across all three cell stages. **c**, Connectivity enrichment analysis. Enrichment of interactors of proteins encoded by genes associated with intellectual disability and autism (NDD) and differentially expressed genes (FDR<5%) in the early developmental stages after 1000 random permutations (empirical permutations testing, one-sided). Gene products encoded by genes linked to inflammatory bowel disease (IBD) yielded no enrichment for protein-protein interactions between the differentially expressed genes at any cell stage, and genes linked to Parkinson's disease (PD) yielded no enrichment in NPCs or neurons.

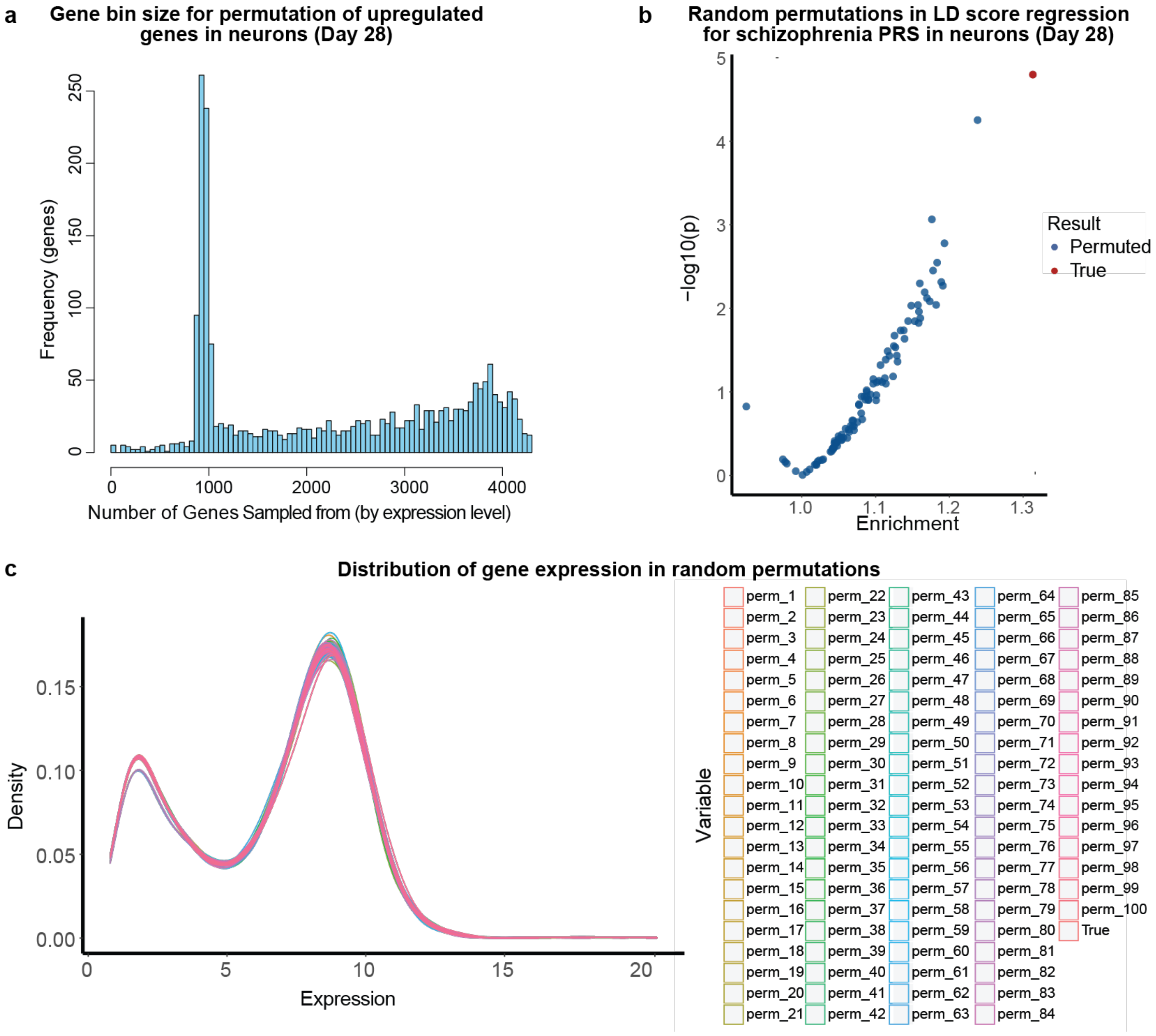

Supplementary Fig. 5. LD-score regression analysis. a, Gene bin size for permutation of upregulated genes in neurons. **b**, Heritability enrichment in LD score regression for 100 random permutations (in blue) and the upregulated genes (in red) in neurons **c**, Distribution of gene expression in random generated gene sets in the 100 permutations.

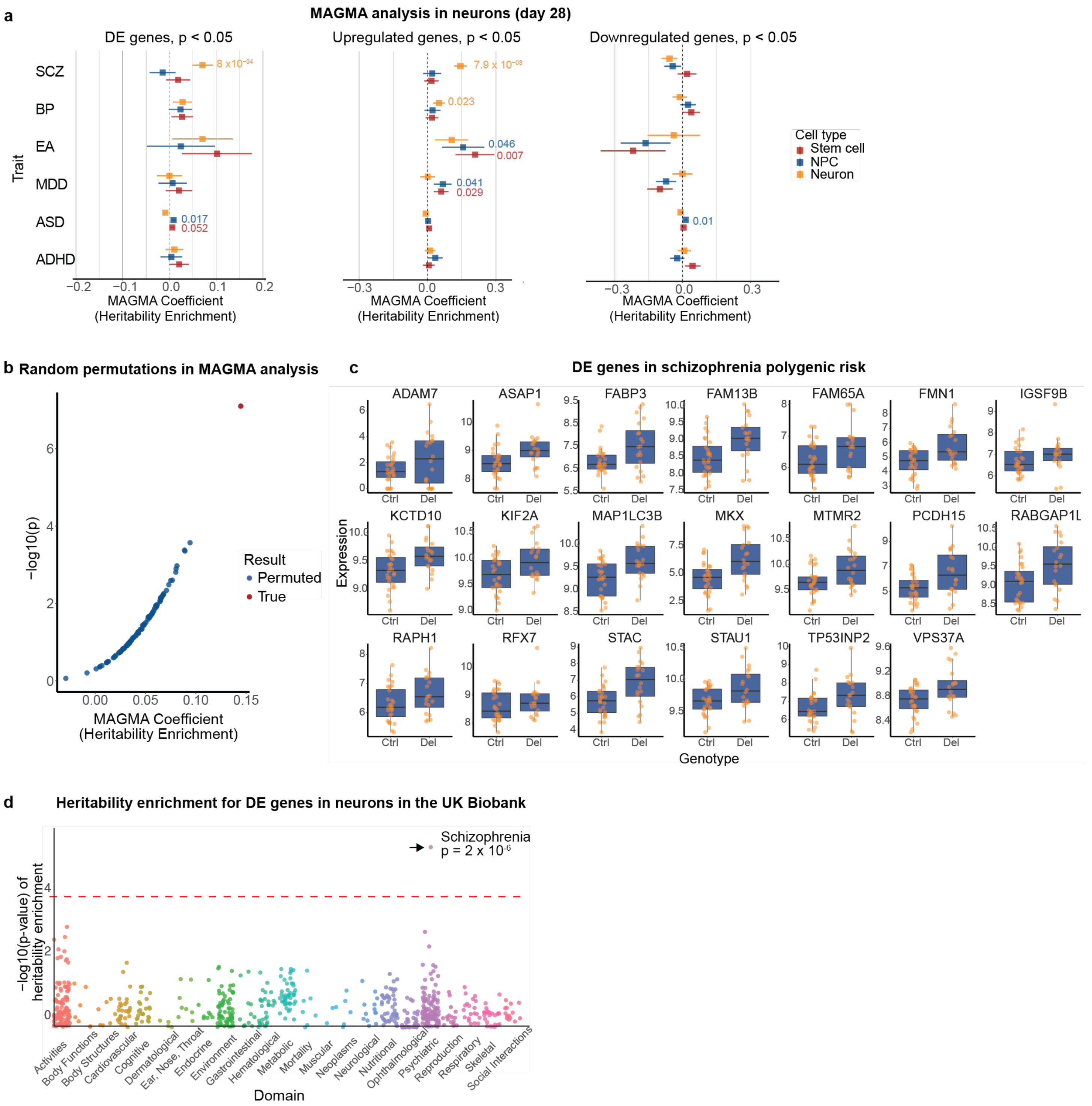

Supplementary Fig. 6. MAGMA (Multi-marker Analysis of GenoMic Annotation) analysis in neurons. a, MAGMA heritability enrichment (presented as the coefficient +/- SE) analysis of six traits across the three developmental cell stages. SCZ= schizophrenia, BP=bipolar disorder, EA= educational attainment, MDD=major depressive disorder, ASD=autism spectrum disorder, ADHD=attention deficit hyperactivity disorder. The number of DE genes that overlapped with gene-wise associations for each trait from MAGMA and the test statistics for each comparison are listed in Supplementary Data 7. **b**, Magma heritability enrichment in random expression matched gene lists from 100 permutations (in blue) compared to the up-regulated genes in neurons (in red).**c,** Expression of the differentially expressed genes contributing to the

MAGMA schizophrenia signal (FDR <5%). (N (patients) = 19, N (controls)=29). The expression values are presented for each cell line in a Tukey style boxplot with the median (Q2) and the first and the second quartiles (Q2, Q3) and error bars defined by the last data point within +/- 1.5-times the interquartile range. **d**, GWAS summary statistics for 650 traits from the UK-biobank showing significant enrichment for heritability only for schizophrenia ($p=2x10^{-6}$) in genes upregulated in deletion neurons in the complete data set N = 20 patients and 29 controls.

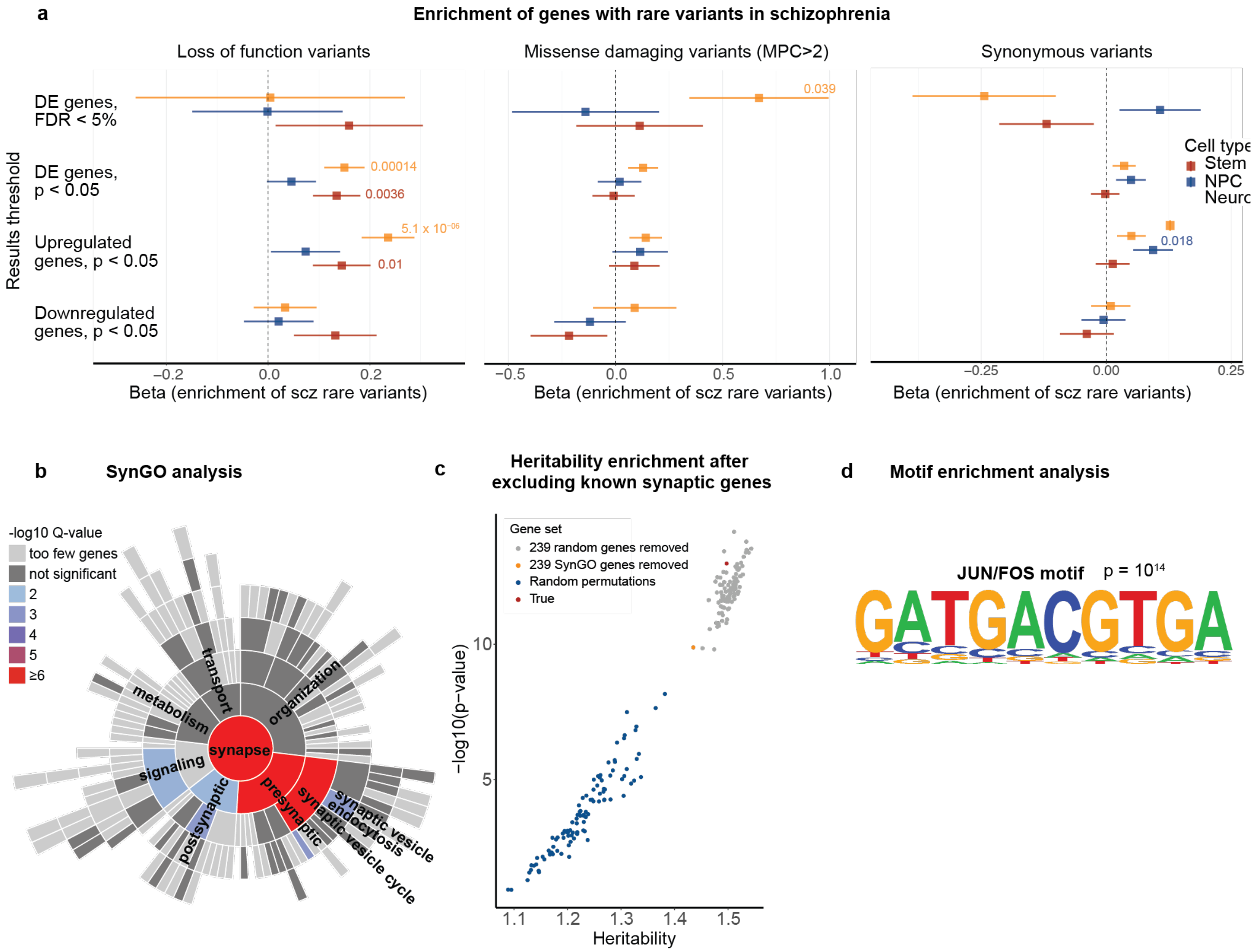

Supplementary Fig. 7. Enrichment analysis of genes with rare variants in schizophrenia, and of genes with synaptic ontologies. a, Forest plots for the difference in number of rare coding variants (presented as coefficient, beta +/- SE) between schizophrenia patients and controls for loss of function, missense damaging and synonymous variants in genes at different significance cutoffs: FDR <5% (N (genes in stem cells) = 212, N (NPCs) = 216, N (genes in neurons) = 86), p <0.05 (all genes; N (genes in stem cells) = 2,346, N (NPCs) = 2,076, N (genes in neurons) = 3,370)), upregulated genes with p <0.05 (N (genes in stem cells) = 1,521, N (NPCs) =964, N (genes in neurons) =2,173) and downregulated genes with p <0.05 (N (genes in stem cells) = 825, N (NPCs) =1,112, N (genes in neurons) = 1,197) at all developmental stages. The test statistics for each comparison are presented in Supplementary Data 8. **b-d**, Analysis including the cell line with the short deletion, SCBB1430. **b**, SynGO annotation for genes upregulated in neurons showing enrichment for synaptic processes (enrichment: 1.4, SE =0.065, $p=1.3\times10^{-10}$, True enrichment in DE genes: 1.5, SE =0.064, $p=1.1\times10^{-13}$, N = 20 patients, 29 controls). **c**, Heritability enrichment for schizophrenia after excluding the 239 genes with SynGO annotation. **d,** Motif Enrichment analysis in upregulated genes (p <0.05), showing enrichment of JUN / FOS targets ($p = 1.0 x10^{-14}$, Homer De novo motif enrichment analysis).

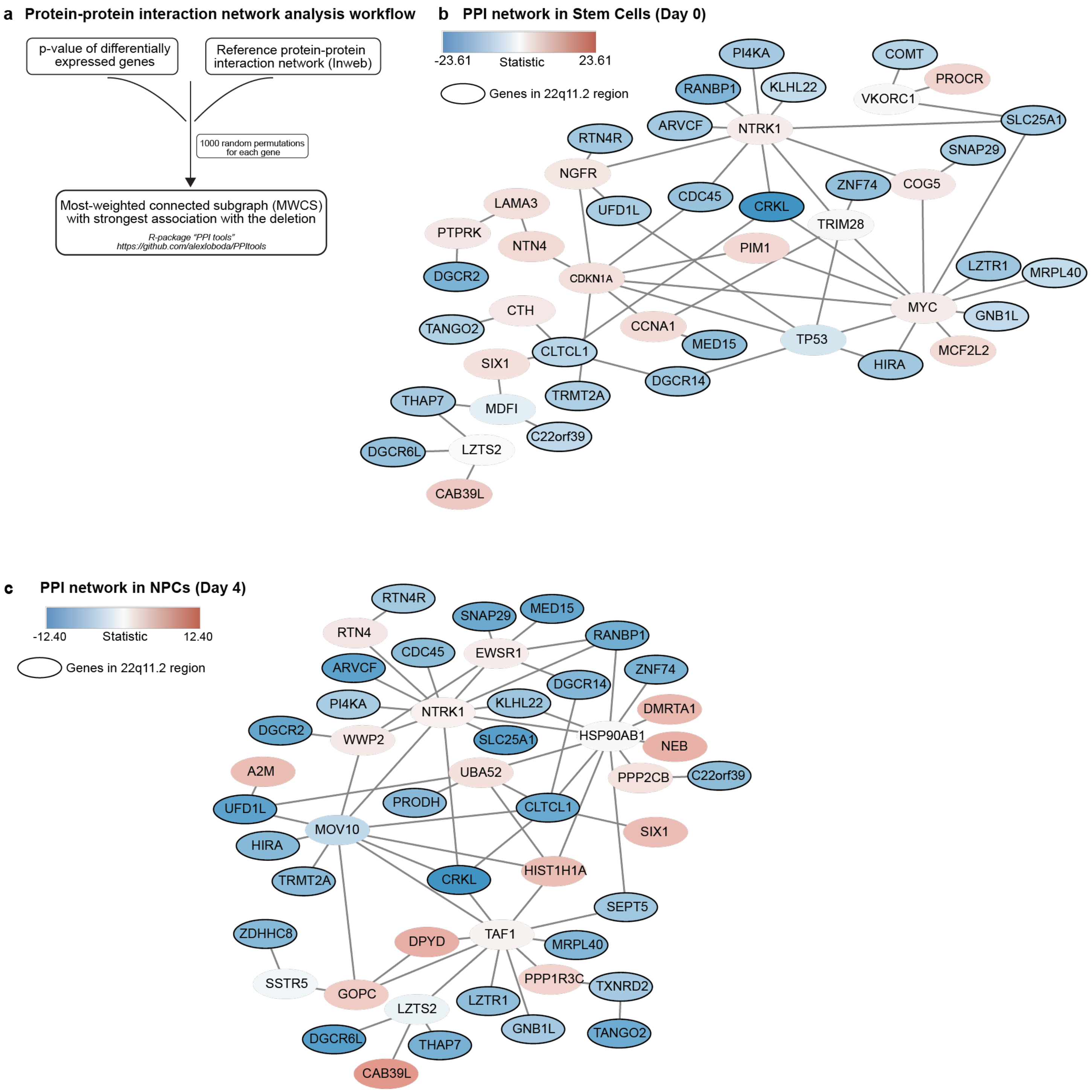

Supplementary Fig. 8. The most weighted sub-cluster graph for protein-protein interactions (PPI) for differentially expressed genes. a, Workflow. **b**, Network in stem cells. **c**, Network in neuronal progenitor cells.

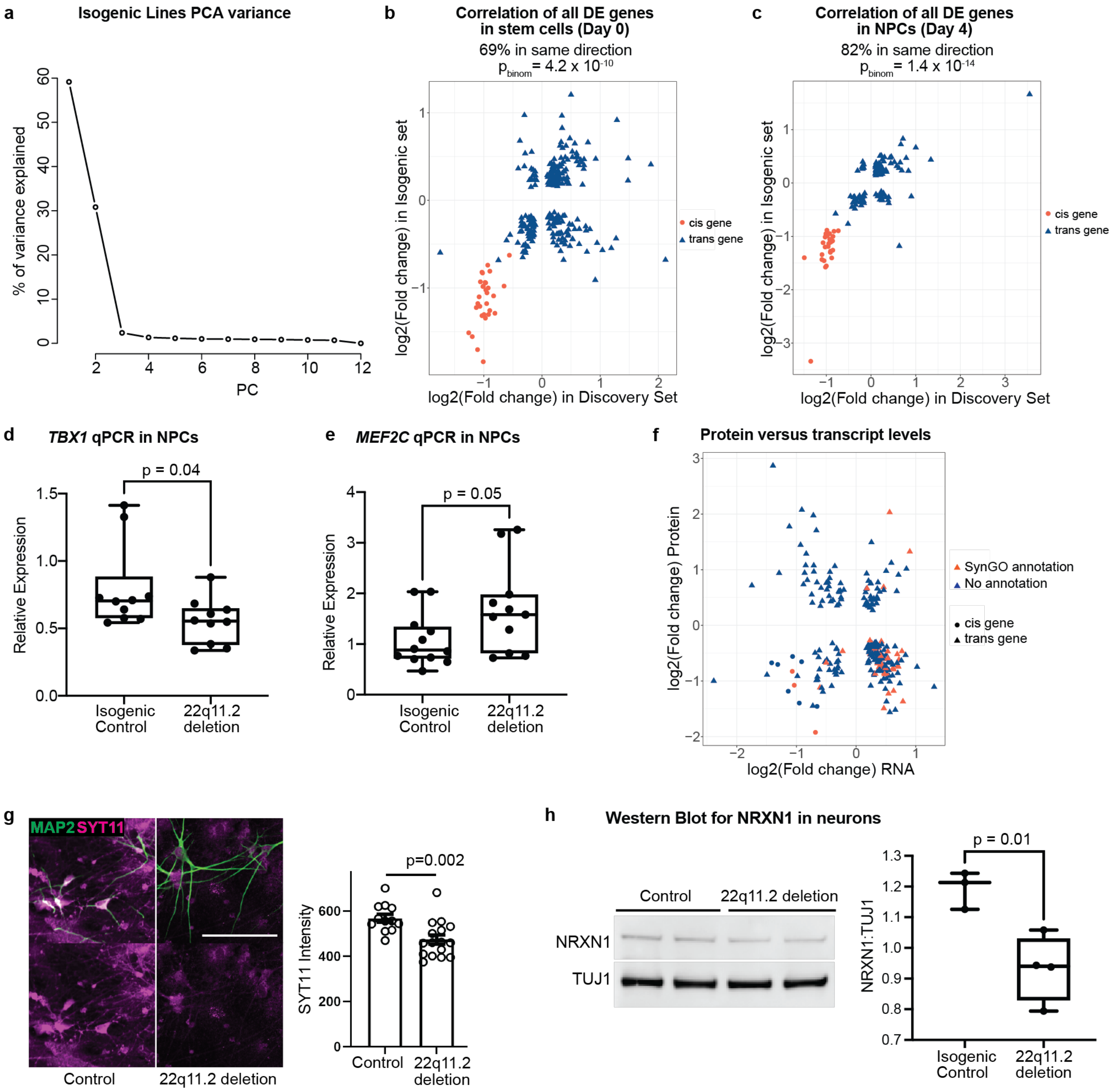

Supplementary Fig. 9. Isogenic line and protein analysis. a, Variance explained by each principal component from RNA sequence data in the isogenic lines. **b-c**, Correlation of fold-changes of differentially expressed genes in discovery and isogenic datasets in stem cells (**b**) and neuronal progenitors (**c**). Red circles = cis genes. Blue triangles = trans genes. **d**, Relative expression of *TBX1* via qPCR in Day 4 NPCs from isogenic control and 22q11.2del lines (N=4, p=0.04, two-sided Student's t test). **e**, Relative expression of *MEF2C* via qPCR in Day 4 NPCs from isogenic control and 22q11.2del lines (N=4, p=0.05, two-sided Student's t test). **f**, Protein versus RNA levels of genes differentially expressed in 22q11.2del carrier patient neurons. Genes with a SynGO annotation are shown in red, genes with no SynGO annotation are shown in blue. Circles = cis genes. Triangles = trans genes. **g**, Synaptotagmin-11 (SYT11) protein levels are decreased in Day 28 22q11.2del neurons. (Left) Representative confocal images of control and 22q11.2del patient neurons stained with antibodies against SYT11 (magenta) and MAP2 (green). Scale bar is 100 µm. (Right) Quantification of total SYT11 fluorescence within MAP2-positive area normalized to controls. Data are represented as means ± SEM. Individual points are

analyzed fields of view from 3 independent control lines and 4 patient-derived lines. Statistical analysis by two-sided Student's t test reveals statistically significant (p=0.0022) decrease in SYT11 levels in patient-derived neurons. **h**, Expression of Neurexin-1 (NRXN1) in total protein lysates from isogenic control and 22q11.2del neurons. (Left) Total protein lysates from isogenic control (left two lanes) and deletion lines (right two lanes) stained for NRXN1 (top) and TUJ1 (bottom). Data are represented as a box plot with individual values superimposed on the graph. Whiskers extend from minimum to maximum value and the box's bounds are 25th and 75th percentile and it is centered in the median. (Right) Statistical analysis by two-sided Student's t test reveals statistically significant decrease in NRXN-1 expression in the deletion lines. (Samples: 5/5, 2BR/2TR, p=0.01). BR = biological replicate (independent differentiations); TR = technical replicate (independent wells).

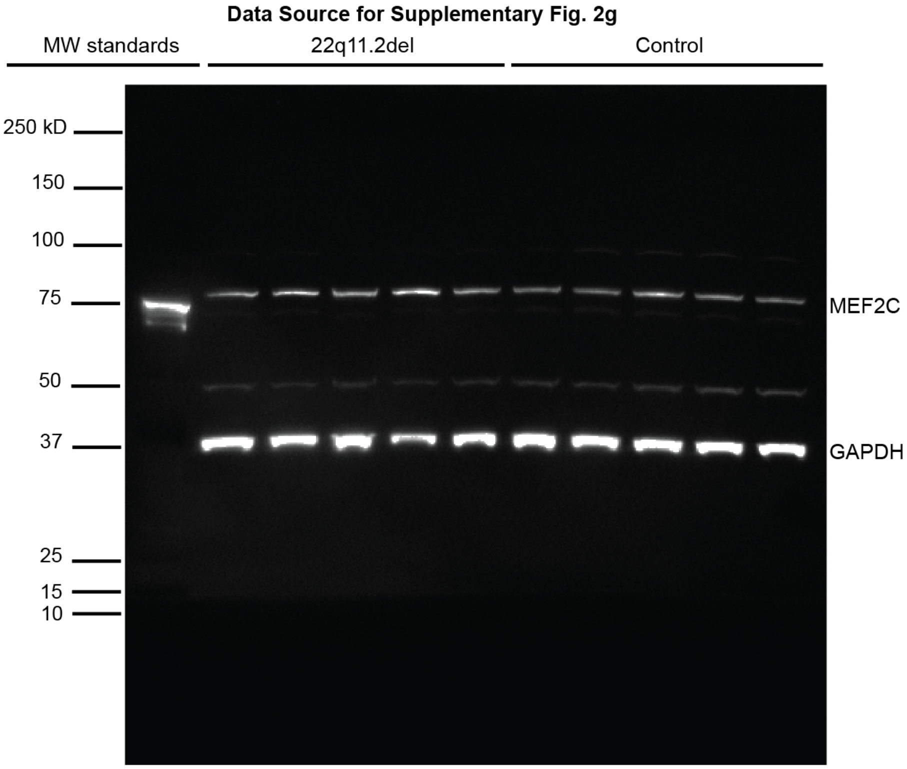

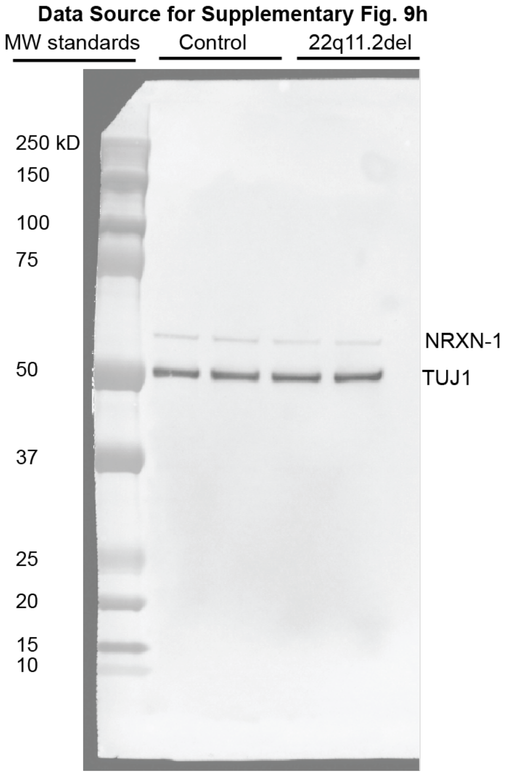

Supplementary Fig. 10. Data source: uncropped gels with molecular weight standards
