# Supplementary material for: The 22q11.2 region regulates presynaptic gene-products linked to schizophrenia
Source: Nat Commun. 2022 Jun 27;13:3690. doi: 10.1038/s41467-022-31436-8 (PMC9237031; doi:10.1038/s41467-022-31436-8)
Supplement: Supplementary file 3 — Description of Additional Supplementary Files [file 41467_2022_31436_MOESM3_ESM.pdf]

Description of Additional Supplementary Files

Supplementary Data 1. Differentially expressed genes at day 0 (22q11.2 deletion vs controls, Wald-test implemented in DESeq2)

Supplementary Data 2. Differentially expressed genes at day 4 (22q11.2 deletion vs controls, Wald-test implemented in DESeq2)

Supplementary Data 3. Differentially expressed genes at day 28 (22q11.2 deletion vs controls, Wald-test implemented in DESeq2)

Supplementary Data 4. Mean expression of cell type specific differentially expressed genes. 95% confidence intervals for mean expression in parenthesis. p-values (Mann-Whitney) presented for the difference in mean expression between the tissues

Supplementary Data 5. List of Disease genes used in Supplementary Fig. 4c

Supplementary Data 6. Results of the LD score regression analysis on genes differentially expressed in 22q11.2 deletion carrier cells (p-values derived from LD score regression)

Supplementary Data 7. Results of MAGMA on genes differentially expressed in 22q11.2 deletion carrier cells (gene set enrichment analysis implemented in MAGMA)

Supplementary Data 8. Results of the rare variant analysis based on data from the SCHEMA consortium in genes differentially expressed in 22q11.2 deletion carrier cells (linear regression)

Supplementary Data 9. Gene Priorities from the Protein-Protein Interaction (PPI) analysis. Results were obtained through empirical permutations testing (one-sided test).

Supplementary Data 10. SYNGO (Synaptic Gene Ontology) enrichment analysis of the genes upregulated at the transcriptional level in neurons (Fisher exact test implanted in SynGO portal)

Supplementary Data 11. Gene Ontology (GO) enrichment analysis of genes upregulated in deletion carrier neurons (Hyper geometric test)

Supplementary Data 12. Gene Ontology (GO) enrichment analysis of genes upregulated in NPCs of deletion carriers (Hyper geometric test)

Supplementary Data 13. Gene Ontology (GO) enrichment analysis of genes downregulated in neurons of deletion carriers (Hyper geometric test)

Supplementary Data 14. JUN/FOS targets identified amongst the genes upregulated in deletion carrier neurons ($p<0.05$)

Supplementary Data 15. Differentially expressed genes at day 0 in isogenic 22q11.2 deletion carriers vs isogenic controls (t-test implemented in limma)

Supplementary Data 16. Differentially expressed genes at day 4 in isogenic 22q11.2 deletion carriers vs isogenic controls (t-test implemented in limma)

Supplementary Data 17. Differentially expressed genes at day 4 in isogenic 22q11.2 deletion carriers vs isogenic controls (t-test implemented in limma)

Supplementary Data 18. SYNGO (Synaptic Gene Ontology) enrichment analysis of the genes upregulated at the transcriptional level in neurons (Fisher exact test implanted in SynGO portal)

Supplementary Data 19. Expression of genes implicated in the ubiquitination pathway (in the discovery cohort) in isogenic lines Wald- test implemented in DESeq2

Supplementary Data 20. Differentially expressed peptides at day 28 (22q11.2 deletion vs controls, t-test implemented in limma)

Supplementary Data 21. SYNGO (Synaptic Gene Ontology) enrichment analysis of the protein level in 22q11.2 deletion neurons (Fisher exact test implanted in SynGO portal)

Supplementary Data 22. Discovery Sample Counts

Supplementary Data 23. Isogenic Sample Counts. Clones A and D are controls, and clones E and G carry the 22q11.2 deletion.

Supplementary Data 24. Total Number of sequenced reads per sample, from the feature Counts output.
