# Supplementary material for: The 22q11.2 region regulates presynaptic gene-products linked to schizophrenia
Source: Nat Commun. 2022 Jun 27;13:3690. doi: 10.1038/s41467-022-31436-8 (PMC9237031; doi:10.1038/s41467-022-31436-8)
Supplement: Supplementary file 28 — Reporting Summary [file 41467_2022_31436_MOESM28_ESM.pdf]

nature portfolio

Corresponding author(s): Ralda Nehme

Last updated by author(s): May 18, 2022

Reporting Summary

Nature Portfolio wishes to improve the reproducibility of the work that we publish. This form provides structure for consistency and transparency in reporting. For further information on Nature Portfolio policies, see our Editorial Policies and the Editorial Policy Checklist.

Statistics

For all statistical analyses, confirm that the following items are present in the figure legend, table legend, main text, or Methods section.

n/a	Confirmed	
☐	☒	The exact sample size (*n*) for each experimental group/condition, given as a discrete number and unit of measurement
☐	☒	A statement on whether measurements were taken from distinct samples or whether the same sample was measured repeatedly
☐	☒	The statistical test(s) used AND whether they are one- or two-sided<br>*Only common tests should be described solely by name; describe more complex techniques in the Methods section.*
☐	☒	A description of all covariates tested
☐	☒	A description of any assumptions or corrections, such as tests of normality and adjustment for multiple comparisons
☐	☒	A full description of the statistical parameters including central tendency (e.g. means) or other basic estimates (e.g. regression coefficient) AND variation (e.g. standard deviation) or associated estimates of uncertainty (e.g. confidence intervals)
☐	☒	For null hypothesis testing, the test statistic (e.g. *F*, *t*, *r*) with confidence intervals, effect sizes, degrees of freedom and *P* value noted<br>*Give P values as exact values whenever suitable.*
☒	☐	For Bayesian analysis, information on the choice of priors and Markov chain Monte Carlo settings
☒	☐	For hierarchical and complex designs, identification of the appropriate level for tests and full reporting of outcomes
☐	☒	Estimates of effect sizes (e.g. Cohen's *d*, Pearson's *r*), indicating how they were calculated

Our web collection on statistics for biologists contains articles on many of the points above.

Software and code

Policy information about availability of computer code

Data collection	No software was used for data collection
Data analysis	Computer code relevant to the PPi analysis has been deposited in GitHub (https://doi.org/10.5281/zenodo.6555972). Other computer code and data analysis will be made available upon request. All analysis was conducted using publicly available tools and software including: STAR (v2.5), Trimmomatic (v0.36), Rsubread (v.1.32), DESeq2, sva (version 3.32), RNASeqPower (vl.18.0), Limma package (v. 3.3.49), MAGMA(v1.06) and ImageJ (v1.52p)

For manuscripts utilizing custom algorithms or software that are central to the research but not yet described in published literature, software must be made available to editors and reviewers. We strongly encourage code deposition in a community repository (e.g. GitHub). See the Nature Portfolio guidelines for submitting code & software for further information.

Data

Policy information about availability of data

All manuscripts must include a data availability statement. This statement should provide the following information, where applicable:

- Accession codes, unique identifiers, or web links for publicly available datasets
- A description of any restrictions on data availability
- For clinical datasets or third party data, please ensure that the statement adheres to our policy

All summary-level data generated in this study are available in Tables S23 and S24. The raw sequence datasets generated for the study are not yet deposited in a public repository due to varied consent provenance within our selected cohort and subsequent data access restrictions. Subsets of the data will be made available by the corresponding authors upon reasonable request within a 30 day timeframe under a data transfer agreement. The following human reference genome was used: ENSEMBL/GRCH37/Hg19 (http://ftp.ensembl.org/pub/grch37/). A reporting summary for this Article is available as a Supplementary Information file.

March 2021

Field-specific reporting

Please select the one below that is the best fit for your research. If you are not sure, read the appropriate sections before making your selection.

☒ Life sciences ☐ Behavioural & social sciences ☐ Ecological, evolutionary & environmental sciences

For a reference copy of the document with all sections, see nature.com/documents/nr-reporting-summary-flat.pdf

Life sciences study design

All studies must disclose on these points even when the disclosure is negative.

Sample size	We used an initial pilot data set of four iPSC lines (two controls and two deletion carriers) to estimate the required sample size for reliable detection of changes in the abundance of transcripts encoded by genes that fall within the deletion interval and whose expression should be reduced by 50% in the deletion carriers. The power estimates were calculated using RNASeqPower12 (R package version 1.18.0). We calculated the median expression and variance in carriers and controls for all genes with one or more reads (25,264 genes) in the pilot data sets. We assumed equal number of cases and controls, while the coefficient of variance was calculated separately for cases and controls. The alpha level was set to 5 x 10-6 assuming Bonferroni correction for 10 000 independent tests. For the final data set the power to detect fold changes of >2 was calculated for each gene separately.
Data exclusions	We initially collected samples from 51 independent donors. However, two of the samples failed library preparation and RNA sequencing, producing low read coverage, resulting in their exclusion from the final dataset (n = 49 samples)
Replication	To ensure the reproducibility of our experimental findings, we 1) used a sufficiently powered sample size (n = 49 iPSC lines derived from unique donors, 2) used three technical replicates for each sample, and 3) engineered the 22q11.2 deletion in control pluripotent stem cell lines using CRISPR / CAS9 to validate the findings in an isogenic setting. Information about how often each additional validation experiment was replicated is included in the figure legend (qPCR, immunostaining, Western Blot, ddPCR and DNA FISH were typically performed at least 3 times and / or on multiple independent cell lines, with all replication attempts being sucessful).
Randomization	This is not relevant to our study. Patient cell lines were defined by the presence of the deletion (based on SNP array data) and unaffected individuals were confirmed to not carry the deletion. All experiments included cells without the deletion as well as cells with the deletion to be able to compare the two groups. All experimental procedures were carried out in batches that included a balance of deletion carriers and controls. Data analysis was performed on the complete final dataset.
Blinding	Investigators were blinded to group allocation during data collection and/or analysis.

Reporting for specific materials, systems and methods

We require information from authors about some types of materials, experimental systems and methods used in many studies. Here, indicate whether each material, system or method listed is relevant to your study. If you are not sure if a list item applies to your research, read the appropriate section before selecting a response.

Materials & experimental systems

n/a	Involved in the study	
☐	☒	Antibodies
☐	☒	Eukaryotic cell lines
☒	☐	Palaeontology and archaeology
☒	☐	Animals and other organisms
☒	☐	Human research participants
☒	☐	Clinical data
☒	☐	Dual use research of concern

Methods

n/a	Involved in the study	
☒	☐	ChIP-seq
☒	☐	Flow cytometry
☒	☐	MRI-based neuroimaging

Antibodies

Antibodies used	The following antibodies were used: rabbit anti-SV2A (1:1000, Abcam ab32942), chicken anti-MAP2 (1:10,000, Abcam ab5392), rabbit anti-Synaptotagmin-11 (1;1000, Synaptic Systems 270 003), mouse anti-hNA (1:1000, Millipore MAB1281), rabbit anti-Cux1 (1:500, Santa Cruz m-222), guinea pig anti-NeuN (1:1000, Synaptic Systems 266004), mouse anti-Sox2 (1:500, R&D systems, MAB2018), mouse anti-Sox1 (1:500, R&D systems, AF3369), mouse anti-Ki67 (1:500, BD Biosciences 550609), rabbit anti-FoxG1 (1:500, abcam ab18259), Neurexin-1 antibody (Millipore ABN161-I, 1:1,000), Tuj1 (Biolegend 801201, 1:5,000), MEF2C (Abcam ab211493, 1:200 dilution, EPR19089-202) and GAPDH (Proteintech, 60004-1-Ig, 1:5000). Alexafluor plus-555 and Alexafluor plus-488 conjugated secondary antibodies (1:5,000) were obtained from Invitrogen.
Validation	All antibodies used in this study are commercially available and have previously been validated.<br><br>- pan-Neurexin1: Millipore-sigma ABN161-I (emdmillipore.com/US/en/product/Anti-pan-Neurexin-1-Antibody,MM_NF-ABN161-I):

From website: Evaluated by Western Blotting in mouse brain tissue lysate.
Western Blotting Analysis: 0.2 µg/mL of this antibody detected Neurexin-1 in 10 µg of mouse brain tissue lysate.

- MEF2C: Abcam, ab211493 https://www.abcam.com/mef2c-antibody-epr19089-202-chip-grade-ab211493.html
From website: this antibody has been validated for Western Blot, and the expression profile observed is consistent with the literature (PMID: 18450586)

- FOXG1: Abcam, ab18259 - 100ug; https://www.abcam.com/foxg1-antibody-ab18259.html
From website: previously published https://pubmed.ncbi.nlm.nih.gov/33993189/

- Nestin: Clone 10C2, unconjugated ; https://www.stemcell.com/anti-human-nestin-antibody-clone-10c2.html
Verified: FC, ICC, IF, WB Reported: FC, ICC, IF, IHC, IP, WB This antibody clone has been verified for labeling human neural stem and progenitor cells grown with STEMdiff™ Neural Induction Medium (Catalog #05835), STEMdiff™ Neural Progenitor Medium (Catalog #05833) and NeuroCult™ NS-A Proliferation Kit (Human; Catalog #05751).

- SOX1 AF3369 R&D systems. From website (https://www.rndsystems.com/products/human-mouse-rat-sox1-antibody_af3369): SOX1 in ectoderm differentuiated BG01V Human Embryonic Stem Cells. SOX1 was detected in immersion fixed BG01V human embryonic stem cells differentiated into neural progenitor cells using Goat Anti-Human/Mouse/Rat SOX1 Antigen Affinity-purified Polyclonal Antibody (Catalog # AF3369) at 10 µg/mL for 3 hours at room temperature. Cells were stained using the NorthernLights™ 557-conjugated Anti-Goat IgG Secondary Antibody (red; Catalog # NL001) and counterstained with DAPI (blue). Nestin was also detected using Mouse Anti-Mouse/Rat Nestin Monoclonal Antibody (Catalog # MAB2736) and stained using the NorthernLights™ 493-conjugated Anti-Mouse IgG Secondary Antibody (green; Catalog # NL009). Specific staining of SOX1 was localized to nuclei.

- Sox2 R&D systems MAB2018; https://www.rndsystems.com/products/human-mouse-rat-sox2-antibody-245610_mab2018
From website: E Cadherin and SOX2 in BG01V Human Stem Cells. E-Cadherin and SOX2 were detected in BG01V human embryonic stem cells using 10 µg/mL Goat Anti-Human E-Cadherin Antigen Affinity-purified Polyclonal Antibody (Catalog # AF648) and 10 µg/mL Mouse Anti-Human/Mouse/Rat SOX2 Monoclonal Antibody (Catalog # MAB2018). Cells were incubated with primary antibodies for 3 hours at room temperature. Cells were stained for E-Cadherin using the NorthernLights™ 557-conjugated Anti-Goat IgG Secondary Antibody (red; Catalog # NL001) and for SOX2 using the NorthernLights 493-conjugated Anti-Mouse Secondary Antibody (green; Catalog # NL009). Cells were counterstained with DAPI (blue).

- Human Nuclear Antigen (hNA) ms millipore MAB1281 clone 235-1; https://www.emdmillipore.com/US/en/product/Anti-Nuclei-Antibody-clone-235-1,MM_NF-MAB1281
Previously published:
Immunofluorescence (IF): A representative lot detected Nuclei in Immunofluorescence applications (Duan, S., et al. (2015). Nat Commun.;6:10068; Saleh, A., et al. (2019). Sci Rep. 9(1):14612; RuizPerera, L.M., et al. (2018). Sci Rep. 8(1):16012).
Immunocytochemistry Analysis (ICC): A representative lot detected Nuclei in Immunocytochemistry applications (Duan, S., et al. (2015). Nat Commun. 6:10068).

- CUX1: SC (M-222) https://www.scbt.com/p/cdp-antibody-m-222
Previously published:
PMID: # 26752160 Carabalona, A. et al. 2016. Nature neuroscience. 19: 253-62.
PMID: # 26043730 Pan, X. et al. 2015. Molecular brain. 8: 36.
PMID: # 25788693 Kohno, T. et al. 2015. J. Neurosci. 35: 4776-87.
PMID: # 25404384 Liu, J. et al. 2014. Molecular brain. 7: 84.
PMID: # 23874761sc-13024 Jeong, S.J. et al. 2013. PloS one. 8: e68781.
PMID: # 24019958 Gan, X.T. et al. 2013. PloS one. 8: e74235.
PMID: # 22209758 Mueller, JK. et al. 2012. Molecular and cellular endocrinology. 351: 184-98.
PMID: # 22223678 Mairet-Coello, G. et al. 2012. Development. 139: 475-487.
PMID: # 22470319 Denham M et al. 2012. Front Cell Neurosci. 6: 11.
PMID: # 21666133 Teissier, A. et al. 2012. Cereb. Cortex. 22: 403-416.

- NeuN: Guinea Pig Synaptic Systems 266 004 https://sysy.com/product/266004
Previously published:
TDP-43 represses cryptic exon inclusion in the FTD-ALS gene UNC13A. Ma XR, Prudencio M, Koike Y, Vatsavayai SC, Kim G, Harbinski F, Briner A, Rodriguez CM, Guo C, Akiyama T, Schmidt HB, et al. Nature (2022) 6037899: 124-130. . ICC; tested species: human

- Map2: Chicken polyclonal ABCAM ab5392
https://www.abcam.com/map2-antibody-ab5392.html
From website: Rat E20 cultured cortical neuron-glial cells stained for MAP2 (red) using ab5392 at 1/10000 dilution for ICC/IF.
Previously published Ahn LY et al. An epilepsy-associated ACTL6B variant captures neuronal hyperexcitability in a human induced pluripotent stem cell model. J Neurosci Res 99:110-123 (2021).PubMed: 33141462
McNutt PM et al. Neuronal delivery of antibodies has therapeutic effects in animal models of botulism. Sci Transl Med 13:N/A (2021).PubMed: 33408188
Xu J et al. MicroRNA expression profiling after recurrent febrile seizures in rat and emerging role of miR-148a-3p/SYNJ1 axis. Sci Rep 11:1262 (2021).PubMed: 33441699
Nilsson F et al. Single-Cell Profiling of Coding and Noncoding Genes in Human Dopamine Neuron Differentiation. Cells 10:N/A (2021).PubMed: 33445654
Wang C et al. ApoE-Isoform-Dependent SARS-CoV-2 Neurotropism and Cellular Response. Cell Stem Cell 28:331-342.e5 (2021).PubMed: 33450186
Li Y et al. Activation of MAP3K DLK and LZK in Purkinje cells causes rapid and slow degeneration depending on signaling strength. Elife 10:N/A (2021).PubMed: 33475086
Trujillo CA et al. Pharmacological reversal of synaptic and network pathology in human MECP2-KO neurons and cortical organoids. EMBO Mol Med 13:e12523 (2021).PubMed: 33501759
Rosenkranz SC et al. Enhancing mitochondrial activity in neurons protects against neurodegeneration in a mouse model of multiple sclerosis. Elife 10:N/A (2021).PubMed: 33565962

Ye T et al. Efficient manipulation of gene dosage in human iPSCs using CRISPR/Cas9 nickases. Commun Biol 4:195 (2021).PubMed: 33580208
Morimoto Y et al. A unique missense variant in the E1A-binding protein P400 gene is implicated in schizophrenia by whole-exome sequencing and mutant mouse models. Transl Psychiatry 11:132 (2021).PubMed: 33602898

Eukaryotic cell lines

Policy information about cell lines

Cell line source(s)

The source of each cell line is listed in Table 1. All induced pluripotent stem cell lines were derived from primary samples (fibroblasts or blood cells) derived from Donors through collaborations with the Northern Finnish Intellectual Disability Collection, Umea University, Karolinska Institute, MGH, GTEx, Stanford University, or McLean Hospital.
Written institutional review board (IRB) approvals and study consent forms from each of the organizations contributing samples were sent to the Broad Institute of Harvard and MIT before the samples were sequenced and analyzed. All relevant ethical guidelines have been followed, and any necessary IRB and/or ethics committee approvals have been obtained. All ethical approvals are on file at the IRB office at Massachusetts General Brigham (MGB), formerly Partners, amended to protocol no. 2016P000058 'Cellular programming for neurobiological disease research'. This approval undergoes annual continuing review by the MGB Human Research Committee IRB. Supplementary review was conducted by the Broad Institute Office of Research Subject Protection.

Authentication

- SNP Genotyping (Infinium Global Screening Array-24 from Illumina; processed at the Genomics Platform, Broad Institute) for cell line identification and detection of chromosomal abnormalities. In brief, we utilize a SNP genotyping pipeline to fingerprint parental cell lines and confirm cell identity at the beginning and end of experiments. We also use data from SNP genotyping run through a modified version of PennCNV to identify CNVs and trisomies that can accumulate over time in stem cell cultures. We submit samples for SNP genotyping to check for chromosomal abnormalities every 8-12 weeks for actively utilized stem cell lines.
- Mycoplasma Testing (MycoAlert, Lonza) for detection of bacterial contamination. We perform mycoplasma testing of cells growing in our incubators on a monthly basis to confirm that our cell culture facilities and individual cell lines are mycoplasma-free.
- Quarantine: We use separate dedicated tissue culture rooms for cell line quarantine (used to house any new incoming cell lines prior to mycoplasma testing) and for lentiviral transduction.
- Reagent Validation: Stem cell reagents (e.g., mTeSR media, small molecules, growth factors) are purchased in bulk and hPSC lines are routinely tested for pluripotency (immunostaining to confirm expression of OCT4, SOX2, SSEA4, TRA-1-60, etc.) and germ layer differentiation capacity (EB formation followed by immunostaining for germ layer markers such as SMA, AFP, TUBB3) as part of our cell culture practices. This allows us to detect changes in the health of our stem cell cultures that may be due to problematic reagents.

Mycoplasma contamination

All cell lines used in this study tested negative for mycoplasma contamination

Commonly misidentified lines
(See ICLAC register)

No commonly misidentified cell lines were used in the study
